# A best–worst scaling experiment to prioritize concern about ethical issues in citizen science reveals heterogeneity on people-level v. data-level issues

**DOI:** 10.1038/s41598-021-96743-4

**Published:** 2021-09-27

**Authors:** Christi J. Guerrini, Norah L. Crossnohere, Lisa Rasmussen, John F. P. Bridges

**Affiliations:** 1 Center for Medical Ethics and Health Policy, Baylor College of Medicine, 1 Baylor Plaza, Houston, TX 77030 USA; 2 Department of Biomedical Informatics, The Ohio State University College of Medicine, 220N Lincoln Tower, 1800 Cannon Drive, Columbus, OH 43210 USA; 3 Department of Philosophy, The University of North Carolina Charlotte, 9201 University City Blvd., Charlotte, NC 28223 USA

**Keywords:** Ecology, Environmental sciences, Medical research, Astronomy and planetary science

## Abstract

“Citizen science” refers to the participation of lay individuals in scientific studies and other activities having scientific objectives. Citizen science gives rise to unique ethical issues that stem from the potentially multifaceted contributions of citizen scientists to the research process. We sought to explore the ethical issues that are most concerning to citizen scientist practitioners, participants, and scholars to support ethical practices in citizen science. We developed a best–worst scaling experiment using a balanced incomplete block design and fielded it with respondents recruited through the U.S.-based Citizen Science Association. Respondents were shown repeated subsets of 11 ethical issues and identified the most and least concerning issues in each subset. Latent class analysis revealed two respondent classes. The “Power to the People” class was most concerned about power imbalance between project leaders and participants, exploitation of participants, and lack of diverse participation. The “Show Me the Data” class was most concerned about the quality of data generated by citizen science projects and failure of projects to share data and other research outputs.

## Introduction

“Citizen science” is an umbrella term that refers to the participation of lay individuals in scientific studies and other activities having scientific objectives^1^. Participants in citizen science projects—called “citizen scientists”—potentially make diverse contributions to these projects, depending on their design and objectives^2,3^. Although there are examples of citizen science projects in the social sciences and humanities, they appear to be most prevalent in the natural sciences and include projects focused on, for example, human health, wildlife, ecology, and natural resources and environments^4,5^. The structure and aims of each citizen science project are unique, but they share an ethos of respect for and optimism about the potential of lay individuals to contribute to scientific understanding^2,6^.

As citizen science approaches in research grow in popularity, attention is focusing on the ethical issues they raise^7–16^. Some issues are new to research ethics and stem from the potentially multifaceted contributions of citizen scientists to the research process (e.g., the potential for projects to overburden participants with unpaid “work” or not provide appropriate attribution to participants^8,9,11,12,14^). Other issues result from citizen science’s challenge to current interpretations of research ethics rules and norms (e.g., the assessment of risks and benefits for participants who both act as traditional research subjects and collect, manage, or analyze data^10,11,14^). Yet another class of issues encompasses known issues in research ethics that are potentially aggravated in citizen science contexts (e.g., the use or availability of data in ways inconsistent with expectations of participants and contributors^13,14^).

In 2017, a workshop funded by the U.S. National Science Foundation (NSF) was held for the purpose of identifying ethical issues faced or created by citizen science projects, defined broadly to encompass projects relevant to any scientific field^17^. Titled “Filling the ‘Ethics Gap’ in Citizen Science,” the interdisciplinary workshop, which was attended by almost 40 U.S.-based citizen science project leaders, participants, and scholars, resulted in a master list of over 60 ethical issues. One objective of the workshop was to begin prioritizing these issues^17^. However, the perspectives and experiences of participants were so diverse that it was necessary to expand the time allotted for discussion to ensure that all issues and voices were heard, understood, and taken into account. By the time discussion had concluded, there was insufficient time remaining in the schedule to conduct prioritization activities.

The purpose of this study was to pick up where the workshop left off and begin the work of prioritizing key ethical issues in citizen science. The study collected data using a survey instrument that included a best–worst scaling (BWS) experiment. BWS is a probabilistic discrete choice model grounded in random utility theory, which assumes that when people are asked to make repeated choices, their choice frequencies give an indication of how much they value the items under consideration^18^. There are three kinds (or “cases”) of BWS experiments. In a BWS case 1 experiment, the items (called “objects”) are organized into subsets and respondents select the objects that represent the extremes of a latent, subjective continuum, such as best–worst or most-least^18^. The relative importance of each object can then be estimated from respondents’ selections in choice sets. Because of the advantages of BWS experiments over more conventional methodologies, such as rating and ranking exercises^19–23^, they are used by researchers in many different disciplines to measure levels of concern about, for example, public policy issues^24,25^, consequences of behaviors^20^, use of technologies^26^, risks^21^, and—most relevant to this study—social and ethical issues^27^.

Here, we report data collected from a BWS case 1 experiment that builds on previous efforts to categorize and prioritize ethical issues in citizen science. Given the preliminary and exploratory nature of those efforts, this study was designed to generate rather than test hypotheses. More generally, it demonstrates the utility of stated preference approaches to help prioritize the policy attention of citizen science leaders and promote ethical practices in citizen science.

## Methods

Best practices for using stated preference methods include conducting qualitative research to identify experimental elements and following standards for quantitative rigor in designing the experiment and analyzing the results^28–31^. This study was conducted in accordance with these best practices as well as specific guidance for BWS experiments^18,19,23,32,33^. Survey procedures were approved by Baylor College of Medicine’s Institutional Review Board (Protocol H-42996).

### Object identification, pretesting, and pilot testing

Survey development steps are shown in Supplementary Figure 1. The initial identification of objects for this experiment was based on the master list of ethical issues identified at the NSF-funded workshop. Next, a targeted literature review of English-language journal articles (identified from searches of SCOPUS and PubMed using terms related to “citizen science” and “ethics” in title-abstract fields) was conducted to triangulate this list with ethical issues raised by scholars and to identify potential gaps. In an iterative process similar to that described in prior research^26^, domains for issues were identified and their relationships were characterized in an effort to define domains with minimal overlap. This process resulted in a candidate list of objects describing 11 ethical issues.

To ensure content validity, or the extent to which an experiment takes account of all things deemed important to the relevant constructs^34^, pretesting of the 11 ethical issues as potential objects in the BWS experiment was conducted by telephone with five U.S.-based expert stakeholders. Pretesters were selected based on their leadership of citizen science organizations, leadership of citizen science projects, and/or relevant contributions to citizen science literature. During pretesting cognitive interviews, the salience of each issue and the accuracy and clarity of its draft description were probed. Pretesters were also asked whether other ethical issues should be considered as potential objects in the survey. Based on this feedback, the ethical issues were revised and expanded to 13.

A BWS experiment was then designed and embedded in a pilot survey, which was programmed in Qualtrics. The pilot survey included open-ended items asking respondents about their experience participating in the experiment and whether any part of the survey was confusing or offensive. In addition, respondents were asked to indicate if they would be willing to participate in a follow-up interview.

An invitation to participate in the pilot survey, including a direct link to the survey, was sent by email to all participants in the NSF-funded workshop. Fifteen individuals completed the pilot survey, 8 indicated their willingness to participate in a follow-up interview, and 5 completed a post-pilot interview. In these interviews, pilot survey participants were probed about their experience participating in the pilot survey and their specific responses to open-ended items. They also were invited to provide additional comments about the scope and presentation of the survey. Based on quantitative analysis and the qualitative feedback provided in the open-ended survey items and during post-pilot interviews, the survey was revised and the number of objects was decreased to 11.

### Final survey design and fielding

The final survey, which was also programmed in Qualtrics, is reproduced in the Supplementary Materials. The first section described the survey, eligibility requirements to participate, and the voluntary nature of participation. To participate in the survey, respondents were required to be at least 18 years old and able to complete an English-language online survey. Respondents provided informed consent to participate by clicking on the arrow to proceed with the survey. The requirement of consent was not waived. However, the Institutional Review Board waived the requirement for written documentation of informed consent, consistent with applicable U.S. federal regulations, because the risks to respondents were minimal and the study involved no procedures for which written consent is normally required outside of the research context.

The second section defined citizen science for purposes of the survey and asked respondents about their experiences relevant to citizen science. The third section provided a description of each object. Following each description, respondents were asked whether the ethical issue that the object described was concerning to them. The purpose of these attitudinal items was to encourage respondents to reflect on the object descriptions and provide a means for assessing convergent validity, or the extent to which experimental results are consistent with other measures believed to measure the same construct^34^.

The fourth section comprised the BWS case 1 experiment. The survey used a balanced incomplete block design comprising 11 choice sets and 5 objects per choice set, with each object occurring and co-occurring with other objects the same number of times^35^. All objects were phrased in the negative to avoid response bias due to directionality of phrasing. Table 1 lists the 11 objects presented during the experiment. The underlying latent, subjective continuum was degree of concern, where each choice set was introduced by the question: “Which ethical issue in citizen science causes you the most and least concern?” At the conclusion of the experiment, respondents were asked to identify what factors they took into account when completing the BWS choice sets. The final section of the survey consisted of demographic items.

**Table 1:** Objects in BWS experiment.

Category^†^	Object	Detailed description	Evidence base^‡^
Science	Poor data quality	The quality of data collected and analyzed by projects might be poor. For example, data might be inaccurate because they were collected using improper techniques or were falsified or fabricated	a,b ^8,9,11,12,14^
	Conflicts of interest	Citizen scientists might have undisclosed conflicts of interest that bias their contributions. For example, citizen scientists might have political or financial relationships with organizations that could affect their participation	a,b ^8,11,14^
Risks	Physical harm	Citizen scientists might be physically harmed as a result of their participation in projects. For example, citizen scientists might be injured while collecting data or performing experiments	a ^11^
	Loss of privacy	Citizen scientists might experience a loss of privacy as a result of their participation in projects. For example, their address, relationships, or habits might be intentionally or unintentionally disclosed on the internet	a,b ^10,36^
	Exploitation	Projects might take advantage of their citizen scientists. For example, projects might overburden citizen scientists with work or require unreasonable amounts of time or money to participate	a,b ^8,11,14^
	No intellectual property	Projects might not respect the intellectual property interests of citizen scientists or their communities. For example, projects might require citizen scientists to give up their intellectual property rights as a condition of participating	a,b ^11,16^
	Conflicting expectations	Projects might use data or findings in ways that conflict with the expectations of citizen scientists or their communities. For example, projects might share data with individuals whom citizen scientists did not expect would have access to data	a,b ^13,14^
Returns	No return of results	Projects might not give citizen scientists or their communities access to study data, findings, or conclusions. For example, projects might not inform citizen scientists of findings that could be relevant to their communities	a,b ^14^
	No credit	Projects might not give credit to citizen scientists or their communities. For example, projects might not acknowledge the contributions of participants or communities on project websites or in publications	a,b,c ^8,9,12,14^
Inclusion	Lack of diversity	Projects might not recruit citizen scientists from diverse populations or it might be difficult for citizen scientists from diverse populations to participate. For example, online projects might not be accessible to individuals without access to the internet	a ^37^
	Power imbalance	Projects might not provide citizen scientists or their communities meaningful opportunities to be involved in important decisions. For example, projects might exclude citizen scientists from participating in decisions regarding project design, governance, or use of results	a,b,c ^14^

^†^Objects and detailed descriptions were presented to survey respondents. Objects were conceptualized as falling into four categories, which were not presented to respondents: scientific integrity of citizen science projects; potential risks to citizen scientists from project participation; potential returns to citizen scientists from project participation; and structural features of projects relevant to inclusion.

^‡^a = Identified during NSF-funded workshop. b = Endorsed by at least one pretester (excluding comments indicating general endorsement of all objects) during cognitive interviews; endorsements volunteered and specifically solicited. c = Endorsed by at least one pilot survey respondent (excluding comments indicating general endorsement of all objects) in free-text responses or during post-pilot interviews; endorsements volunteered but not specifically solicited. Relevant references published before and after survey development provided in brackets.

The final survey was fielded from January 9, 2020 to March 15, 2020. Respondents were recruited with the help of the Citizen Science Association (CSA), a membership-based organization dedicated to promoting and supporting the efforts of citizen science practitioners and participants^38^. The CSA is based in the United States, but it does not restrict membership by country of residence. In January and February 2020, CSA included an invitation to participate in the survey and a direct link to the survey in its newsletters. In addition, an invitation and direct link were posted to CSA’s discussion listserv twice in February 2020.

### Data analysis

All data analysis was conducted with STATA IC (StatCorp, College Station, TX). BWS scores were calculated by subtracting the number of times an object was selected as least concerning from the number of times it was selected as most concerning and then dividing each count by the total number of times the object appeared during the experiment. Conditional logistic analysis was conducted using a sequential best–worst assumption according to which respondents are assumed to have first selected the most concerning object, followed by the least concerning object, in each choice set. Finally, preference heterogeneity was explored using latent class analysis. Based on fit statistics for 2, 3, and 4 classes, we concluded that the 2-class model was the best fit for the data. Both the conditional logit and latent class models used effects coding, which results in positive and negative coefficients. A positive coefficient represents that the concern was more preferred than the mean; a negative coefficient represents that the concern is less preferred^26^. Importance scores for the conditional logit model were calculated and a probability re-scaling procedure was used to convert scores to a range from 0 to 100^39^.

## Results

In total, 108 respondents made selections for choice sets in the BWS experiment. Respondents were predominantly female (67%), aged 30–59 (60%), and resided in the United States (63%). Two-thirds of respondents (66%) stated that they had participated in a citizen science project in the previous five years and over half (55%) had served as project leaders (54%).

The count method of analysis, which subtracts the “worst” count for each object from the “most” count for that object (in this study, least-concern count was subtracted from most-concern count), generates a BWS score for each object that indicates the relative level of concern associated with it. As reported in Table 2, BWS scores for all respondents show that the four most concerning ethical issues were failure to return results, power imbalance, exploitation, and poor data quality, and the four least concerning ethical issues were no intellectual property, physical harm, no credit, and conflicts of interest. Importance scores generated from conditional logit coefficients indicate that the most concerning ethical issue (no return of results) was approximately twice as influential as the least concerning ethical issue (no intellectual property).

**Table 2:** BWS scores and aggregate and latent class conditional logit models.

Object	BWS score^a^	Aggregate model (*N* = 108)			Latent class model				
					Power to the people (*n* = 41, 38%)		Show me the data (*n* = 67, 62%)		*P*_class 1 = class 2_^c^
		Coefficient	SE	Importance score^b^	Coefficient	SE	Coefficient	SE	
No return of results	0.16	0.440**	0.07	12.40	− 0.030	0.12	0.783**	0.10	< 0.001
Power imbalance	0.13	0.351**	0.07	11.62	1.680**	0.13	− 0.300**	0.09	< 0.001
Exploitation	0.13	0.366**	0.07	11.75	1.221**	0.13	− 0.039	0.09	< 0.001
Poor data quality	0.13	0.289*	0.09	11.10	− 1.656**	0.12	1.594**	0.10	< 0.001
Lack of diversity	0.05	0.125	0.07	9.79	0.538**	0.14	− 0.075	0.10	< 0.001
Conflicting expectations	0.01	0.028	0.07	9.07	0.201	0.13	− 0.082	0.09	0.57
Loss of privacy	− 0.04	− 0.090	0.07	8.25	− 0.122	0.12	− 0.106	0.09	0.84
Conflicts of interest	− 0.09	− 0.224**	0.06	7.38	− 0.398**	0.11	− 0.209*	0.09	0.26
No credit	− 0.13	− 0.327**	0.07	6.77	− 0.518**	0.13	− 0.344**	0.09	0.16
Physical harm	− 0.14	− 0.446**	0.08	6.11	− 0.546**	0.15	− 0.495**	0.11	0.10
No intellectual property	− 0.20	− 0.512**	0.07	5.78	− 0.374**	0.11	− 0.726**	0.09	0.26

^a^BWS scores calculated by subtracting least-concern count for each object from its most-concern count and dividing by the number of times the object appeared in the survey (5 × *N*).

^b^Importance scores calculated by rescaling coefficients from conditional logit on a ratio scale from 0 to 100.

^c^Wald test of overall model differences: *P* < 0.001.

*P $$\le$$ 0.01, **P $$\le$$ 0.001.

**Figure 1:**
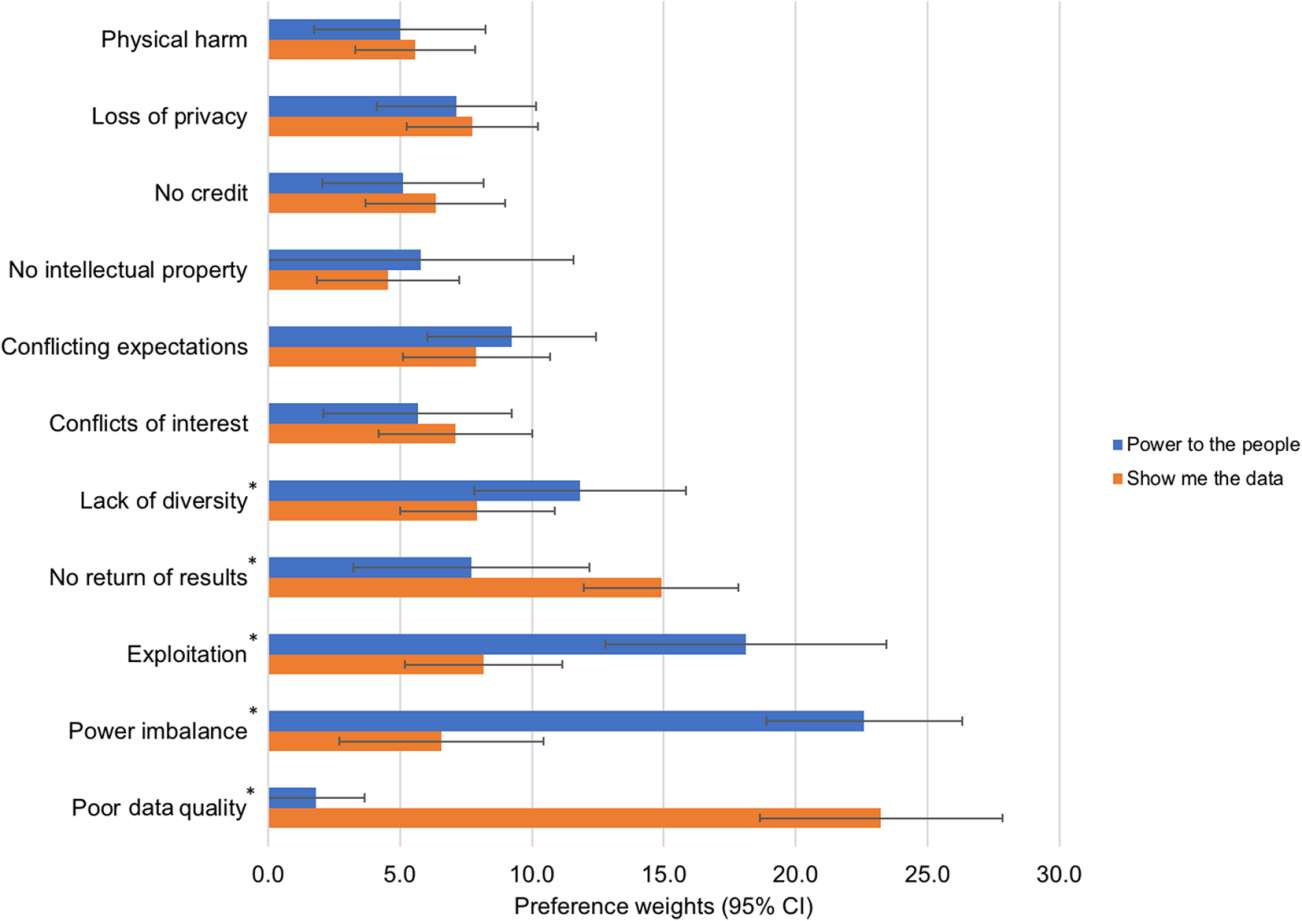
Latent class logit model. *Between groups comparison, *P*$\hspace{0.17em}\le \hspace{0.17em}$0.05.

However, as shown in Table 2 and Fig. 1, latent class analysis identified heterogeneity of preferences clustered in two groups, where prioritization estimates for five ethical issues were significantly different between these groups. The first class, which contained 38% of respondents, is labeled “Power to the People” and focused on ethical issues related to who participates in citizen science projects, what are their roles, and whether the privileges, opportunities, and burdens of their participation are equitably distributed. The second class, which contained 62% of respondents, is labeled “Show Me the Data” and focused on ethical issues related to quality of and access to data generated in citizen science projects.

Specifically Power to the People class members were most concerned about power imbalance, exploitation, and lack of diversity. Perhaps notably, poor data quality was least concerning to members of this class. As shown in Table 3, compared to Show Me the Data class members, a greater proportion of Power to the People class members were ages 30–59; had led or participated in citizen science conferences and workshops; identified as academics who study citizen science; and contributed to natural resource and human health projects. By contrast, Show Me the Data class members were most concerned about poor data quality and no return of results. Compared to Power to the People class members, a greater proportion of Show Me the Data class members were under age 30 and over age 59.

**Table 3:** Respondent characteristics by class (*N* = 108).

	Power to the people Percent (*n* = 41, 38%)	Show me the data Percent (*n* = 67, 62%)	*P*_class 1 = class 2_
*Age*			0.02
18–29	9.5%	14.3%	
30–59	77.0%	58.0%	
60+	13.5%	27.7%	
Female gender	64.9%	75.6%	0.81
*Race/ethnicity (all that apply)*			
White	86.5%	78.6%	0.17
Hispanic/Latinx	2.7%	6.8%	0.21
Other	5.4%	6.8%	0.75
Country of residence: USA	65.7%	70.0%	0.54
*Roles relevant to citizen science in past 5 years (all that apply)*^a^			
Project participant	60.0%	69.4%	0.15
Conference or workshop participant	72.5%	51.4%	0.002
Project leader	52.5%	56.2%	0.59
Author of citizen science article/commentary	61.0%	47.5%	0.16
Member of citizen science organization	56.5%	45.1%	0.28
Academic who studies citizen science	57.5%	35.4%	0.001
Conference or workshop organizer	50.0%	31.9%	0.01
Leader of citizen science organization	19.5%	23.9%	0.57
*Activities conducted for citizen science projects in past 5 years (all that apply)*^a^			
Collecting data	67.5%	52.7%	0.025
Giving a public presentation	71.2%	40.9%	< 0.001
Publicizing project	56.2%	42.5%	0.04
Recruiting participants	56.2%	39.2%	0.01
Analyzing data	55.0%	39.2%	0.02
Designing research plan	48.8%	38.2%	0.11
Training participants	45.0%	41.4%	0.59
Authoring article/commentary	58.5%	43.3%	0.12
Serving as scientific consultant	28.7%	18.8%	0.07
Serving on community advisory board	18.8%	14.5%	0.39
Donating money	12.5%	9.7%	0.49
Donating physical resources	21.2%	9.7%	0.01
Playing online game	23.8%	7.5%	< 0.001
Serving as non-scientific consultant	14.6%	13.4%	0.86
*Scientific focus of projects (all that apply)*			
Non-human animals	40.0%	38.2%	0.78
Natural resources	55.0%	29.6%	< 0.001
Plants	32.5%	23.7%	0.13
Human health	30.0%	9.1%	< 0.001
Archival	10.0%	9.1%	0.83
Space	12.5%	4.8%	0.03
Human behavior	10.0%	4.3%	0.07
Other	20.0%	8.6%	0.01

^a^Selection of “Other” excluded from analysis.

Latent class analysis results are generally consistent with class-specific responses to attitudinal items preceding the BWS experiment that asked respondents whether the ethical issue that the object described was concerning to them, providing some evidence of convergent validity. As reported in Supplementary Table 1, a greater proportion of Power to the People class members (compared to Show Me the Data class members) were concerned about 10 of the 11 ethical issues presented in the survey. The single exception was poor data quality, which was also the only issue that was concerning to fewer than half of Power to the People class members.

At the conclusion of the BWS experiment, respondents were asked what factors they considered when making selections in choice sets. As reported in Supplementary Table 2, personal experience was most frequently considered (83%) when selecting most and least concerning ethical issues. By contrast, only 21% of respondents considered another person’s experience.

## Discussion

As citizen science becomes more prevalent, increasing attention is being paid to understanding and developing frameworks for addressing the unique ethical issues associated with this research approach. Other research efforts have been directed towards systematically cataloging and prioritizing ethical issues for further consideration. These efforts include expert co-creation of a comprehensive list of issues^17^, expert syntheses of key issues and considerations^8,14,15^, and empirical studies to understand the perspectives of participants and scientific collaborators on project-specific and domain-specific issues^9,36^.

We sought to build on these efforts by reporting the results of an exploratory study to assess relative concern about ethical issues in citizen science. The results indicate that, relative to other ethical issues, the following four ethical issues were most concerning to aggregated respondents: failure to return results, power imbalance, exploitation, and poor quality data. However, the importance of these concerns varied across two underlying respondent classes: one class that was most concerned about the diversity and fair and respectful treatment of participants in citizen science, and a second, relatively larger class that was most concerned about citizen science project data. Further, members of the Power to the People class were generally concerned about more ethical issues than Show Me the Data class members.

Preference heterogeneity might have been driven by age differences between the classes, where individuals age 60 and older comprised over twice the proportion of Show Me the Data class members. This might indicate that older respondents believed that instances of low diversity, power imbalance, and exploitation are infrequent in citizen science or that those instances are relatively less concerning than other ethical issues. However, we believe it is more likely that preference heterogeneity was driven by the possibly broader engagement of Power to the People class members in citizen science projects and the study of citizen science. Indeed, as shown in Table 3, a greater proportion of Power to the People class members participated in every one of the 14 project activities for which we collected data. Greater involvement in projects—especially projects in which participants engage by name and perhaps also in person—can heighten awareness of potential inequities related to who are the participants and what are their experiences of participation. Another potential explanation for preference heterogeneity is the more frequent involvement of some individuals specifically in collaborative or community-based citizen science projects, perhaps co-designed and co-executed by community members. These projects are known to pay close attention to issues such as power dynamics, respect for locally situated knowledge and capabilities, and benefits to communities^40–42^. We did not collect data to test this hypothesis but encourage its study in future research that more thoroughly probes the kinds of projects in which respondents participate in or lead.

Given that Power to the People class members were very concerned about almost every ethical issue in attitudinal items, the experimental results demonstrate the advantages of using stated preference methods over rating models. Rating models do not require trade-offs and therefore do not necessarily reveal preferences^19^ and also are associated with over-selection of the extreme ends of scales^21^. Ranking models, on the other hand, do not assess the magnitude of difference of importance between items^20^ and can be cognitively demanding as the number of items in a set increase^21^. The results of this BWS experiment are therefore richer, more complete, and more likely to reflect true preferences than information that might have been generated using these more conventional approaches.

More generally, this study demonstrates the utility of stated preference approaches to help prioritize the policy attention and efforts of citizen science leaders and practitioners. For example, CSA is collaborating on a grant-funded initiative to build trustworthy data practices for citizen science that focus on five topics: achieving data openness, crediting volunteers, providing return of results, conveying transparency, and respecting data privacy^43^. Our experiment covered three of these issues and found that failure of projects to return data, findings, and conclusions to citizen scientists or their communities is 1.5 and 1.8 times more concerning to stakeholders than, respectively, privacy issues and credit issues. Given that resources to develop tools to support good data practices are limited, the study demonstrates an evidence-based path to prioritizing these and other data tools for citizen science. Separately, our results suggest that similar efforts focused on issues of power and diversity would likely be welcomed.

## Limitations

This study is subject to limitations. First, the experiment was designed to prioritize 11 ethical issues, which represents a significant reduction of the number of issues identified during the NSF-funded workshop. However, best practices for stated preference studies recommend reducing respondent burden^28^ and the number of objects included in this study was consistent with the median for BWS case 1 experiments identified in a recent literature review^44^. Further, the number of included objects was limited by the study’s scope: only ethical issues that potentially might impact any citizen science project, regardless of scientific discipline, were considered for inclusion as objects. Thus, ethical issues known to be of serious concern to specific citizen science projects—for example, disclosure of the location of endangered species in conservation projects^36^ and interference with bodily autonomy interests in human health projects^45–47^—were excluded from consideration. This study was intended to be the first of what will hopefully be many empirically driven efforts to understand and prioritize ethical issues in citizen science, and for that reason, it was considered appropriate for the survey to be inclusive of all projects. However, we encourage the development of discipline-specific and project-specific objects for use in future research designed to identify the ethical issues that are most salient for affected communities.

Second, the sample was a convenience sample and therefore it is not possible to report a response rate or to make inferences based on the relationship of the sample to the sampling frame. The sampling approach was driven by the practical impossibility of developing a diverse, international sampling frame comprised of known citizen science stakeholders and their contact information. However, CSA’s membership, from which the sample was drawn, is known to approximate the U.S.-based target sampling frame. Relatedly, the strategy of recruiting respondents through the CSA newsletter and discussion listserv likely resulted in participation by citizen science leaders whose views about these issues might have been biased by preexisting organizational or institutional commitments. On the other hand, their views are presumably informed by rich experiences in citizen science and understanding of the relevant issues and so might be especially valuable inputs if these data are used to shape policy agendas.

Third, the final sample included a small number of respondents (*n* = 7) who had not actively participated in a citizen science project in the previous five years but engaged in this space as, for example, scholars who studied citizen science. We did not exclude these respondents based on the assumption that they were knowledgeable about citizen science given their connections to CSA and viewed the topic under study as interesting or important given that they were not compensated for participating.

Fourth, the sample size for analysis was relatively small, although it was in the range of sample sizes for recent health care-related BWS case 1 experiments identified by a systematic review^44^. Fifth, pretesting and piloting the survey with U.S.-based individuals resulted in selection and presentation of objects that reflect Western cultural and social norms. Further, recruitment through the CSA resulted in participation primarily by U.S.-based individuals, although approximately one third of respondents resided outside the United States. Due to limited resources we were unable to design and execute a global study, although we support future research focused on capturing non-U.S. perspectives.

## Conclusion

Using a BWS experiment, we sought to explore the ethical issues that are most concerning to citizen scientist practitioners, participants, and scholars to support ethical practices in citizen science. Relative to other ethical issues, the following four ethical issues were most concerning to aggregated respondents: failure to return results, power imbalance, exploitation, and poor quality data. However, the importance of these concerns varied across two underlying respondent classes: one class that was most concerned about the diversity and fair and respectful treatment of citizen science participants and a second class that was most concerned about citizen science project data. This study demonstrates the utility of stated preference approaches to help prioritize the policy attention and efforts of citizen science leaders and practitioners. Going forward, we encourage the development of studies using these approaches for specific projects to better understand and help projects navigate the unique ethical issues associated with their work.

## Supplementary Information

Supplementary Information.

## Data Availability

The data sets generated during the study are available from the corresponding author upon reasonable request.
