# Supplementary material for: A best–worst scaling experiment to prioritize concern about ethical issues in citizen science reveals heterogeneity on people-level v. data-level issues
Source: Sci Rep. 2021 Sep 27;11:19119. doi: 10.1038/s41598-021-96743-4 (PMC8476613; doi:10.1038/s41598-021-96743-4)
Supplement: Supplementary file 1 — Supplementary Information. [file 41598_2021_96743_MOESM1_ESM.docx]

# **Title: A best-worst scaling experiment to prioritize concern about** ethical issues in citizen science reveals heterogeneity on people-level v. data-level issues

**Authors:** Christi J. Guerrini, JD, MPH^1*^, Norah L. Crossnohere, PhD^2^, Lisa Rasmussen, PhD^3^, John F.P. Bridges, PhD^2^

**Affiliations:**

^1^ Baylor College of Medicine, Center for Medical Ethics and Health Policy, 1 Baylor Plaza, Houston, TX 77030.

^2^ The Ohio State University College of Medicine, Department of Biomedical Informatics, 220N Lincoln Tower, 1800 Cannon Drive, Columbus, OH 43210

^3^ The University of North Carolina Charlotte, Department of Philosophy, 9201 University City Blvd., Charlotte*,*NC*28223*.

* Correspondence to: [guerrini@bcm.edu](mailto:guerrini@bcm.edu)

**Supplementary Materials**

Supplementary Fig. 1. Steps to identify and describe BWS experimental objects

| Supplementary Table 1. Endorsement of concern about object in response to attitudinal items (*N*=108) | | | |
| --- | --- | --- | --- |
| Object | Power to the People percent  (*n*=41, 38%) | Show Me the Data percent  (*n*=67, 62%) | *P* |
| No return of results | 88.8% | 81.2% | 0.05 |
| Lack of diversity | 87.5% | 73.4% | 0.30 |
| No credit | 82.5% | 73.4% | 0.19 |
| Power imbalance | 95.0% | 61.7% | <0.001 |
| Poor data quality | 46.2% | 89.4% | <0.001 |
| Conflicting expectations | 72.5% | 60.9% | 0.07 |
| Exploitation | 83.8% | 51.6% | <0.001 |
| No intellectual property | 82.5% | 50.8% | <0.001 |
| Loss of privacy | 76.2% | 44.5% | <0.001 |
| Physical harm | 53.8% | 50.8% | 0.80 |
| Conflicts of interest | 50.0% | 47.7% | 0.91 |

| Supplementary Table 2. Factors considered when making selections (all that apply) (*N*=101) | |
| --- | --- |
|  | Percent |
| My personal experience with issue | 83% |
| Consequences of the issue for project | 60% |
| Difficulty of resolving issue | 45% |
| Number of projects effected by issue | 28% |
| Another person’s personal experience with the issue | 21% |
| Other^a^ | 18% |
| ^a^ Free text responses included consequences for participants, consequences for citizen science as a field, consequences for society, and lack of awareness of the issue | |

**Survey**^[[1]](#footnote-1)^

We are inviting you to participate in a research study being conducted by Christi Guerrini, JD, MPH and Amy McGuire, JD, PhD at Baylor College of Medicine; John Bridges, PhD, at The Ohio State University; and Lisa M. Rasmussen, PhD, at The University of North Carolina at Charlotte.

Participation in this study includes filling out an online survey about ethical issues in citizen science. We will also ask you some questions about yourself, including your age, education, and activities relevant to citizen science.

You must be at least 18 years old to take this survey.

This survey will take approximately 15 minutes to complete. Your participation is completely voluntary and you may stop at any time for any reason.

If you are willing to participate, please click the forward arrow below.

This section asks about your activities relevant to citizen science.

The definition of **citizen science** is evolving.

For purposes of this survey, we define citizen science broadly as any endeavor having a scientific purpose in which individuals who may or may not have scientific training participate as volunteers in one or more activities other than (or in addition to) allowing others to collect and analyze their personal data or specimens. We refer to these volunteer participants as **citizen scientists**.

We distinguish citizen science from **traditional science**, in which participants allow others to collect and analyze their personal data or specimens for research purposes but are not otherwise involved in the research process.

**In the past 5 years, what activities have you participated in relevant to citizen science?** Please select all that apply.


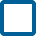
 Leader of a citizen science project


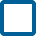

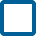
 Participant in a citizen science project Leader of a citizen science organization


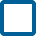
 Member of a citizen science organization

Organizer of a citizen science conference or workshop
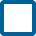
 Participant in a citizen science conference or workshop
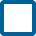
 Academic who studies citizen science


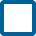


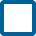
 Author of a citizen science article or commentary
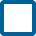
 Other (please specify):


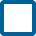
I have not participated in citizen science

# **In the past 5 years, have you participated in one or more citizen science projects?**


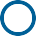
 Yes
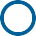
 No

**What activities did you participate in with respect to these projects?** Please select all that apply.


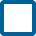
 Designing research plan


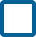
 Recruiting participants


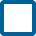
 Training participants
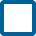
 Publicizing the project
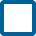
 Collecting data


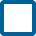
 Analyzing data


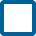
 Playing an online game
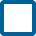
 Donating money


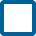
 Donating physical resources (for example: lab space or equipment)
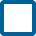
 Serving on a community advisory board


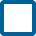
 Serving as a scientific consultant


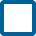
 Serving as a non-scientific consultant (for example: legal or ethics consultant)
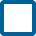
 Authoring an article or commentary


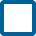
 Giving a public presentation (for example: at a conference)
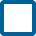
 Other (please specify):

**What was the scientific focus of the projects in which you participated?** Please select all that apply.


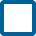
 Archival materials
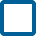
 Human health


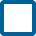
 Human behavior


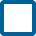
 Other animals (for example: birds or marine mammals)
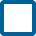
 Plants

Natural resources (for example: water or air)


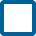


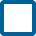
 Space


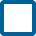
Other (please specify):

The purpose of this next section is to introduce you to 11 ethical issues in citizen science and to understand the extent to which you are concerned about them.

Citizen science projects are diverse. Specific citizen science projects are associated with specific ethical issues. For example, investment in capacity building for communities may be

relevant to environmental justice projects, but not to do-it-yourself health projects. On the other hand, bodily autonomy may be especially relevant to do-it-yourself health projects, but not to environmental justice projects.

The 11 ethical issues that are identified in this survey were selected because they potentially are relevant to any kind of citizen science project.

Some of these 11 ethical issues might also be relevant to traditional science. However, we ask that you think about them only in the context of citizen science.

1. **Poor data quality:** The quality of data collected and analyzed by projects might be poor. For example, data might be inaccurate because they were collected using improper techniques or were falsified or fabricated.

# **Are you concerned about poor data quality in citizen science?**

Yes


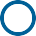


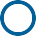
 No


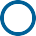
I don't know

1. **Conflicts of interest:** Citizen scientists might have undisclosed conflicts of interest that bias their contributions. For example, citizen scientists might have political or financial relationships with organizations that could affect their participation.

# **Are you concerned about conflicts of interest in citizen science?**

Yes


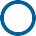


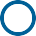
 No


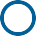
 I don't know

1. **Physical harm**: Citizen scientists might be physically harmed as a result of their participation in projects. For example, citizen scientists might be injured while collecting data or performing experiments.

# **Are you concerned about physical harm in citizen science?**

Yes


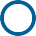


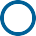
 No


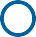
 I don't know

1. **Loss of privacy**: Citizen scientists might experience a loss of privacy as a result of their participation in projects. For example, their address, relationships, or habits might be intentionally or unintentionally disclosed on the internet.

# **Are you concerned about loss of privacy in citizen science?**

Yes


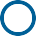


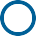
 No


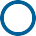
 I don't know

1. **Conflicting expectations:** Projects might use data or findings in ways that conflict with the expectations of citizen scientists or their communities. For example, projects might share data with individuals whom citizen scientists did not expect would have access to data.

# **Are you concerned about conflicting expectations in citizen science?**


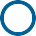
 Yes


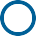
 No


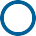
 I don't know

1. **Exploitation:** Projects might take advantage of their citizen scientists. For example, projects might overburden citizen scientists with work or require unreasonable amounts of time or money to participate.

# **Are you concerned about exploitation in citizen science?**

Yes


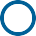


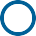
 No


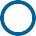
 I don't know

1. **No return of results:** Projects might not give citizen scientists or their communities access to study data, findings, or conclusions. For example, projects might not inform citizen scientists of findings that could be relevant to their communities.

# **Are you concerned about no return of results in citizen science?**

Yes


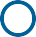


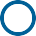
 No


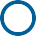
 I don't know

1. **No credit:** Projects might not give credit to citizen scientists or their communities. For example, projects might not acknowledge the contributions of participants or communities on project websites or in publications.

# **Are you concerned about no credit in citizen science?**

Yes


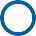


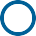
 No


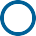
 I don't know

1. **No intellectual property**: Projects might not respect the intellectual property interests of citizen scientists or their communities. For example, projects might require citizen scientists to give up their intellectual property rights as a condition of participating.

# **Are you concerned about no intellectual property in citizen science?**


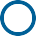
 Yes


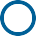
 No


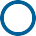
 I don't know

1. **Lack of diversity:** Projects might not recruit citizen scientists from diverse populations or it might be difficult for citizen scientists from diverse populations to participate. For example, online projects might not be accessible to individuals without access to the internet.

# **Are you concerned about lack of diversity in citizen science?**

Yes


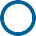


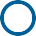
 No


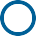
 I don't know

1. **Power imbalance:** Projects might not provide citizen scientists or their communities meaningful opportunities to be involved in important decisions. For example, projects might exclude citizen scientists from participating in decisions regarding project design, governance, or use of results.

# **Are you concerned about power imbalance in citizen science?**

Yes


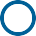


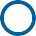
 No


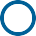
 I don't know

We want to understand the extent to which you are concerned about each of these 11 ethical issues.

We will gauge your concern using what is called a **best-worst scaling** exercise. The exercise consists of 11 questions. Each question will show you a unique subset of the 11 ethical issues and ask you to select the one that causes you the **most** concern and the one that causes you the **least** concern.

This best-worst scaling exercise will take you more time to complete than if we asked you to rate or rank the ethical issues according to your level of concern. However, it will generate richer information, which is why we selected it for this survey.

First, we want to give you a feel for how questions in a best-worst scaling exercise are asked and answered.


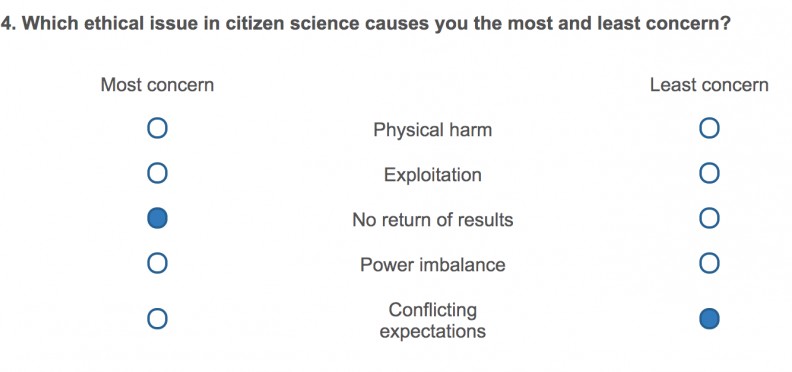
Here's an example of a question that is similar to the ones you will see in a minute.

Let's say that the person who answered this question is named A.J.

A.J.'s answer means that, when asked to consider these 5 ethical issues, no return of results causes

A.J. the **most** concern and conflicting expectations causes A.J. the **least** concern.

Following this same format, we will now ask you about your level of **concern** about ethical issues in citizen science. We will do so through a series of 11 questions.

# **Which ethical issue in citizen science causes you the most and least concern?**


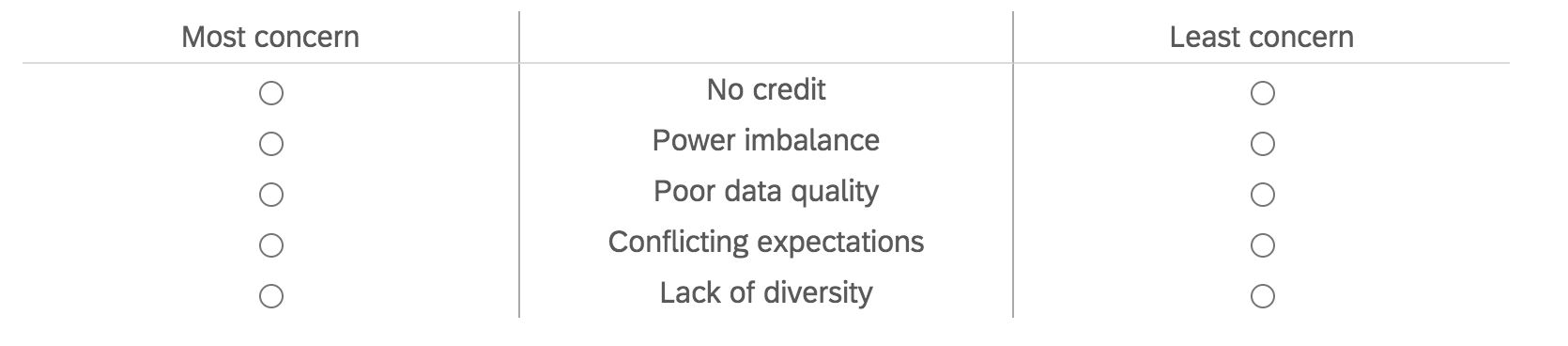


# **Which ethical issue in citizen science causes you the most and least concern?**


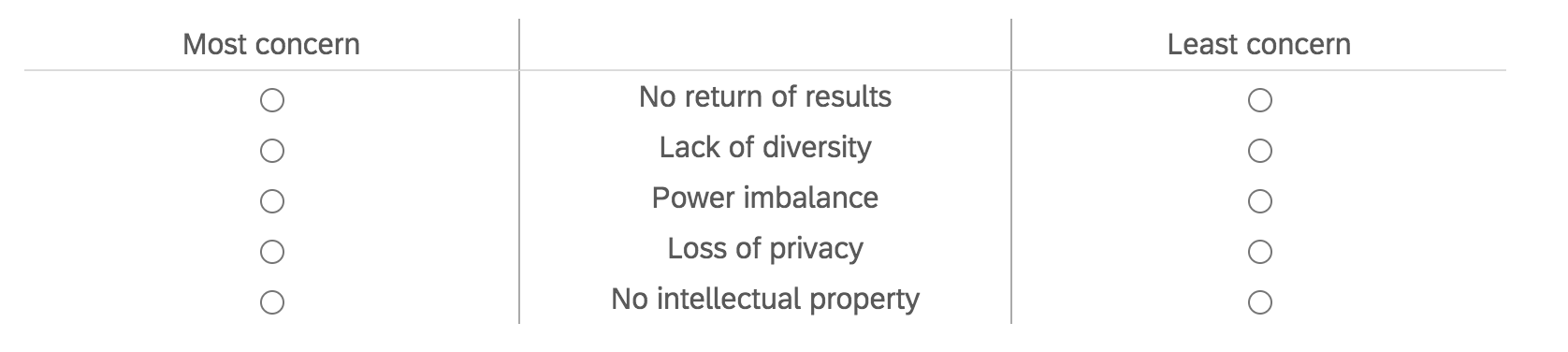


# **Which ethical issue in citizen science causes you the most and least concern?**


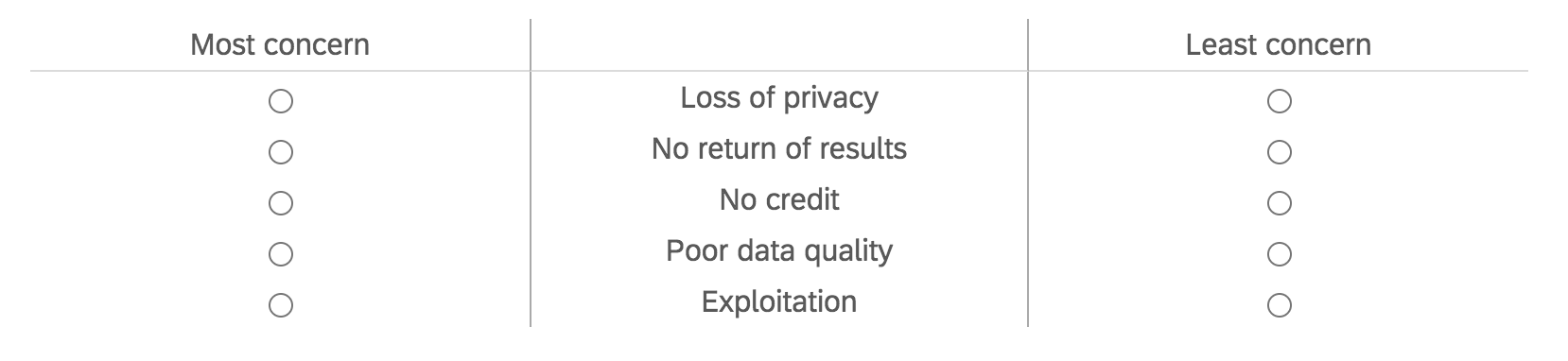


# **Which ethical issue in citizen science causes you the most and least concern?**


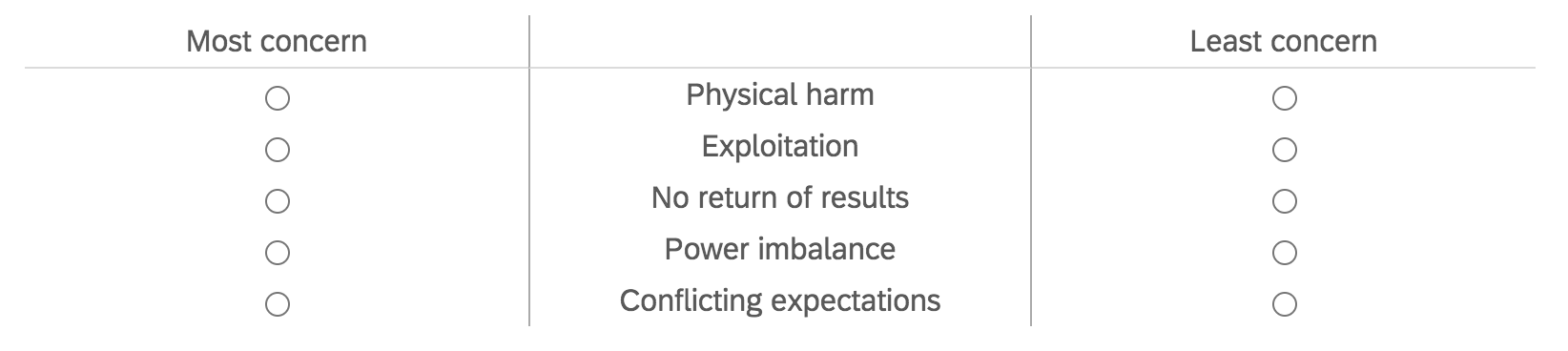


# **Which ethical issue in citizen science causes you the most and least concern?**


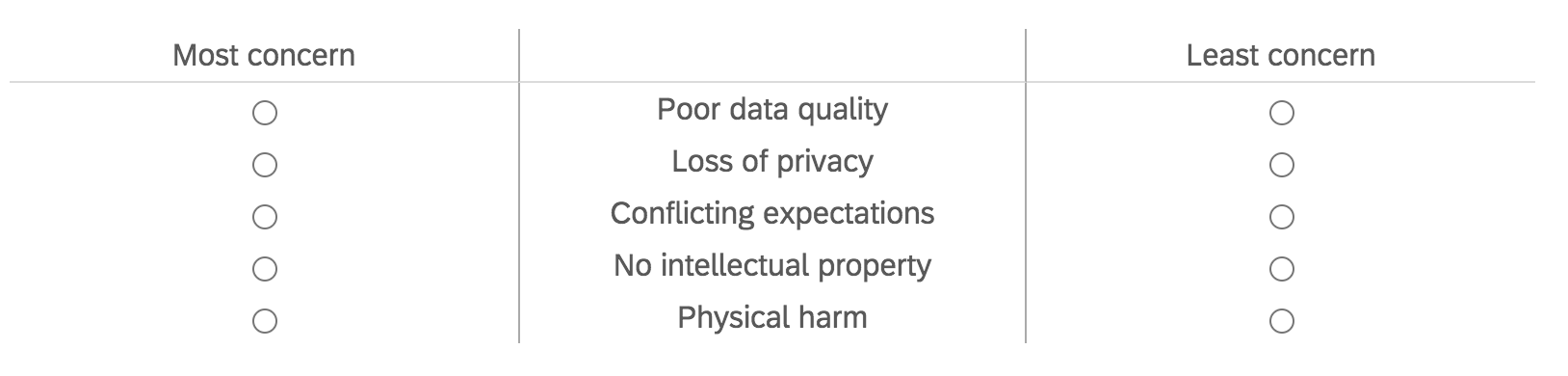


**6. Which ethical issue in citizen science causes you the most and least concern?**


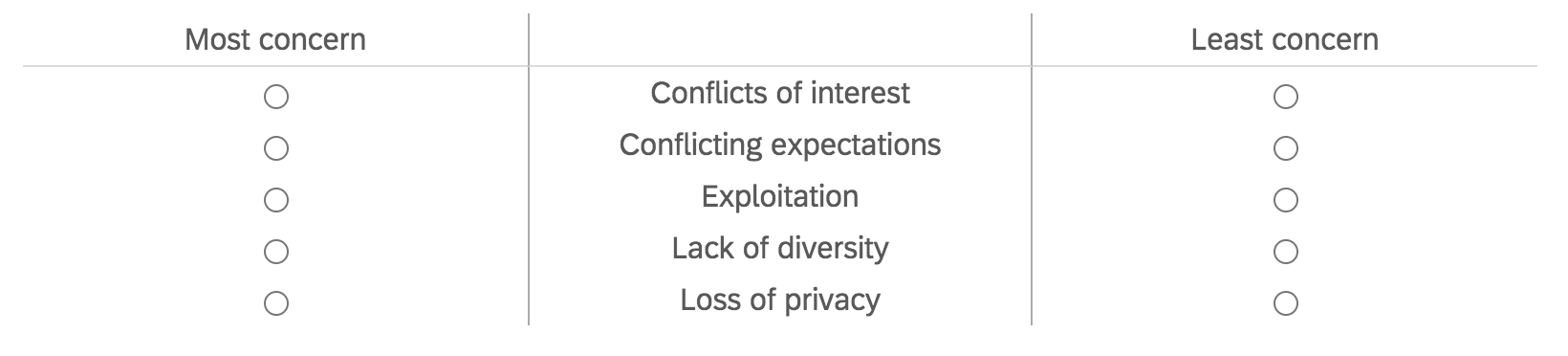


You've completed over half of these questions!

# **Which ethical issue in citizen science causes you the most and least concern?**


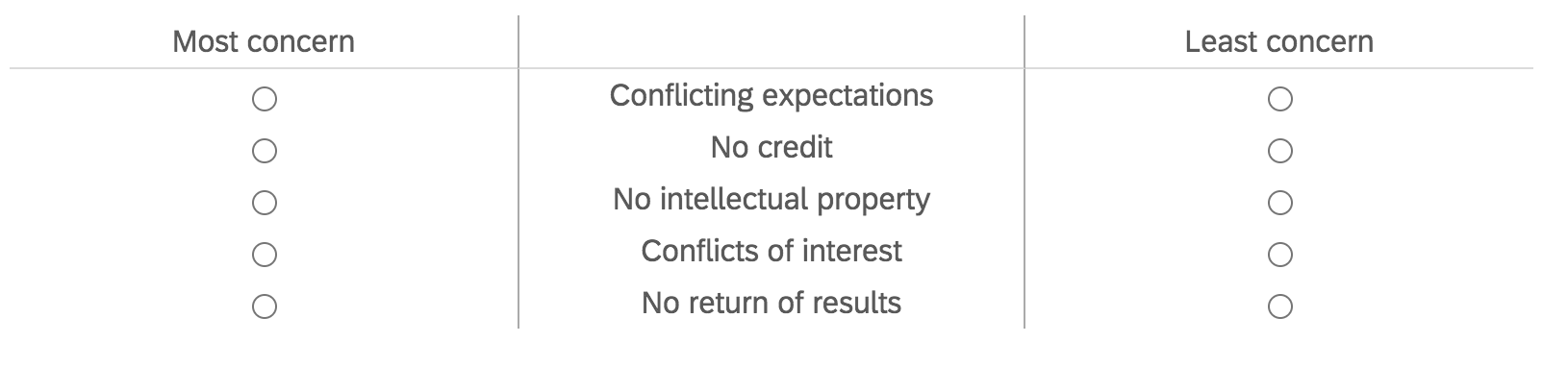


# **Which ethical issue in citizen science causes you the most and least concern?**


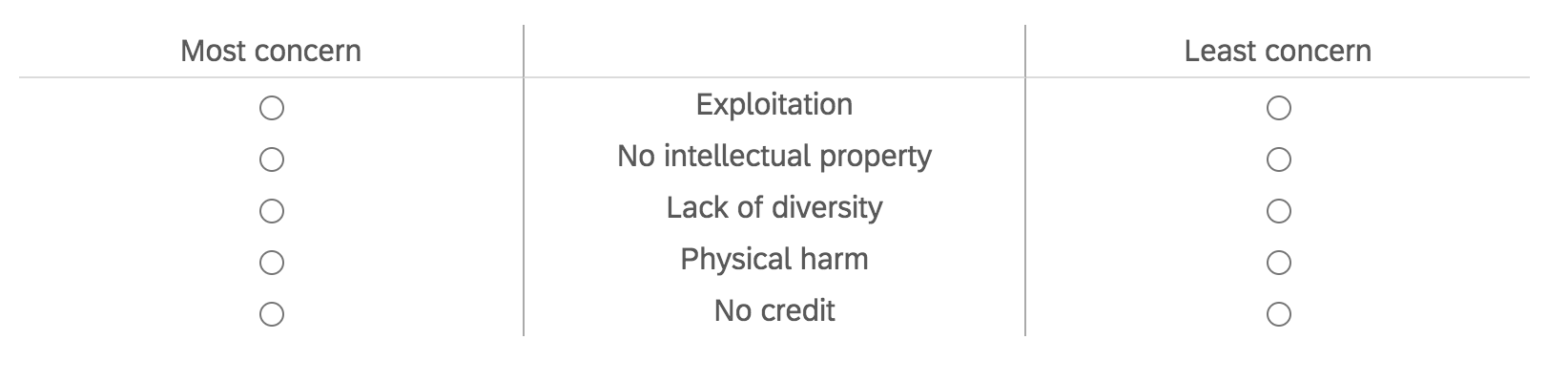


# **Which ethical issue in citizen science causes you the most and least concern?**

**
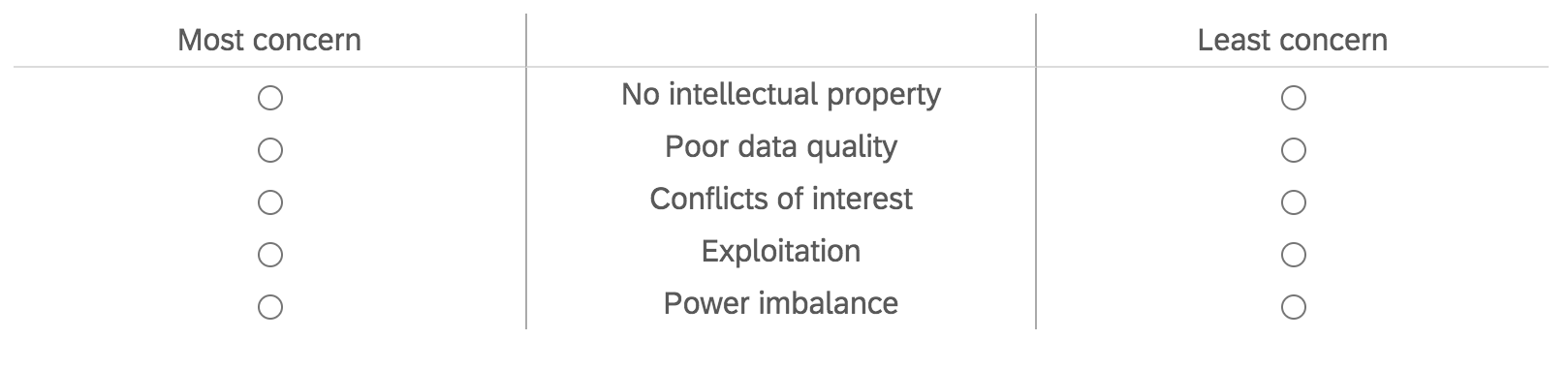
**

# **Which ethical issue in citizen science causes you the most and least concern?**


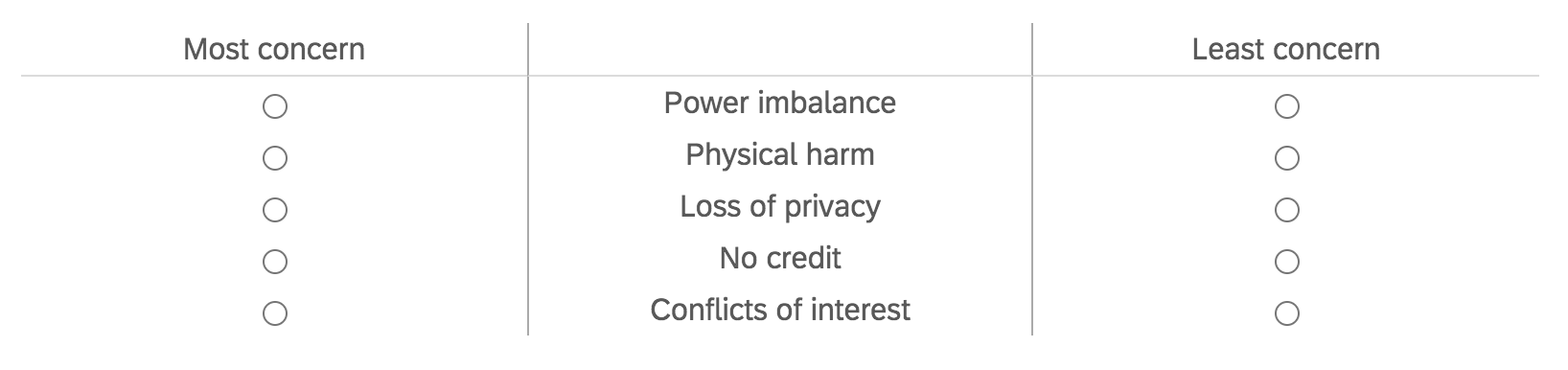


This is the last question in this exercise. Almost done!

# **Which ethical issue in citizen science causes you the most and least concern?**


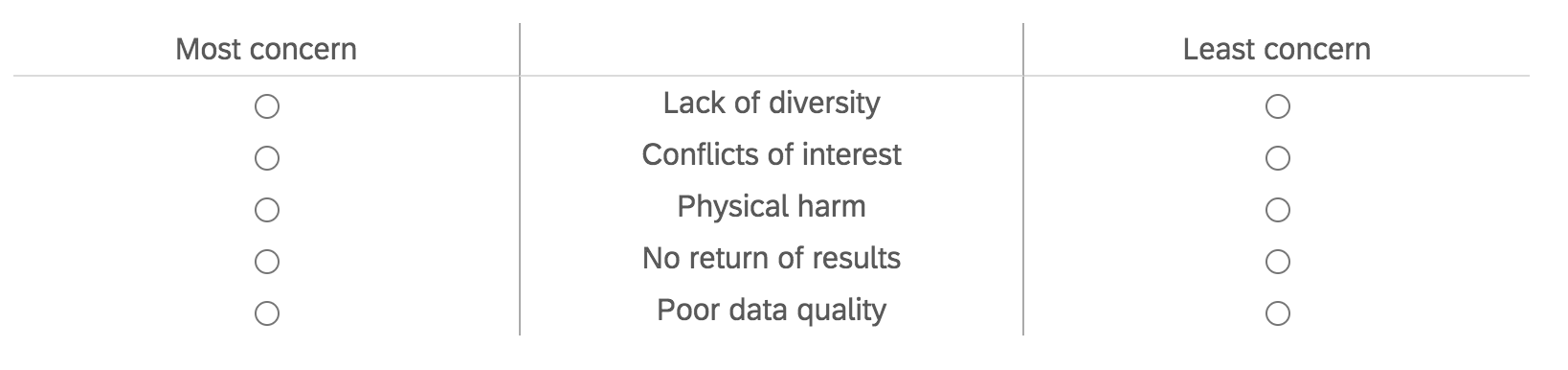


Please consider your responses to the previous 11 questions.

**What factors did you consider when answering the questions?** Please select all that apply.
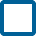
 My personal experience with the issue


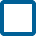
 Another person's personal experience with the issue


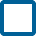

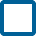
 Number of projects affected by the issue Difficulty of resolving the issue


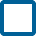

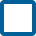
 Consequences of the issue for projects Other (please specify):

**What is your age?**


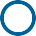
 18-29


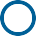
 30-59


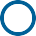
 60 or older

**How do you describe your gender?**


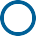
 Male
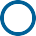
 Female

I prefer to self-describe as (please specify):


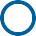


**How do you describe your race/ethnicity?** Please select all that apply.
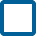
 American Indian, Native American, or Alaska Native


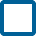
 Asian


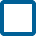
 Black or African American
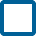
 Hispanic or Latino


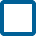
 Middle Eastern or North African/Mediterranean
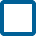
 Native Hawaiian or Pacific Islander


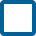
 White or European American

I prefer to self-describe as (please specify):


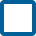


# **In what country do you currently live?**

**In what state or territory do you currently live in the United States?**

# **What is your primary professional discipline?**

Anthropology Archaeology Ecology

Education

Environmental science History

Law

Library science Medicine

Psychology Sociology

Space science Zoology

Other (please specify):

**Is there additional information about your answers that you would like to share?**

# **Are there other ethical issues in citizen science that concern you and you would like to share?**

**Was any part of the survey confusing, incorrect, or offensive?**

# **Did you have any technical difficulties with the survey?**

1. *Skip logic and drop-down options are not shown.* [↑](#footnote-ref-1)
